# Burn Injuries Accelerate Biological Aging and Increase the Epigenetically Inferred Risk of Mortality and Frailty

**DOI:** 10.14336/AD.2025.0407

**Published:** 2026-05-11

**Authors:** Fadi Khalaf, Serena Yang, Dalia Barayan, Diana Julia Tedesco, Michael Chong, Guillaume Paré, Marc G. Jeschke

**Affiliations:** ^1^Department of Biochemistry and Biomedical Sciences, McMaster University, Hamilton, Ontario, Canada.; ^2^David Braley Research Institute, Hamilton, Ontario, Canada.; ^3^Hamilton Health Sciences, Hamilton, Ontario, Canada.; ^4^Department of Surgery, McMaster University, Hamilton, Ontario, Canada.; ^5^Population Health Research Institute, David Braley Cardiac, Vascular and Stroke Research Institute, Hamilton, Ontario, Canada.; ^6^Department of Pathology and Molecular Medicine, McMaster University, Hamilton, Ontario, Canada; ^7^Department of Medicine, McMaster University, Hamilton, Ontario, Canada.

**Keywords:** aging, trauma, burns, frailty, mortality, DNA methylation

## Abstract

Biological aging is closely associated with heightened disease risk, frailty, and mortality. Interestingly, physical traumas, such as burn injuries, exhibit physiological effects that resemble those of aging. However, the impact of burn injuries on biological aging remains underexplored, creating a gap in the literature that could inform better prognosis and outcomes. We conducted a prospective cohort study to investigate the effects of burn injuries on various epigenetic clocks, including HorvathAge, GrimAge, PhenoAge, and DunedinPoAm, using whole blood. The study included 59 burn patients and 25 healthy controls and was validated using a murine model of thermal injury. Our study demonstrates that burn injuries accelerate biological aging and the rate of aging, with these effects persisting for up to 28 days post-injury. The extent of biological aging was positively correlated with burn size, with severe burns resulting in an acceleration of 13-14 years in biological age as measured by GrimAge and PhenoAge-double the acceleration observed with chronic long-term smoking. This acceleration occurred irrespective of age or sex, though older patients were the most vulnerable to the aging effects of burn injuries. The role of burns as an accelerator of aging was further confirmed in mice, which exhibited the equivalent of 3-6 human years of accelerated aging (8 mouse months, or 7 human days) after the injury, reinforcing the chronic nature of the effect. Additionally, burn injuries increased epigenetically inferred risks of frailty and mortality in humans, highlighting their long-term and enduring consequences. Collectively, our findings identify burn injuries as the most significant and chronic accelerant of biological aging reported to date. To our knowledge, this study is one of the first to link burn injuries—or any form of physical trauma—to accelerated cellular and biological aging.

## INTRODUCTION

Biological aging refers to the progressive loss of physiological integrity over time, a process intricately linked to increased disease risk, frailty, and mortality. This deterioration is reflected by molecular hallmarks, which include epigenetic modifications. Significantly, it has become evident that epigenetic age, defined as the age estimate derived from a mathematical algorithm based on the methylation state of specific CpGs in the genome, or “epigenetic clocks,” stands out as the most promising estimator of biological age among potential biomarkers [1]. Indeed, as we age, our genomes undergo gradual and predictable DNA methylation (DNAm) changes, a process commonly referred to as epigenetic drift. While some of these changes are undeniably influenced by hereditary factors, research indicates that epigenetic age can transiently increase in response to extreme life stressors, disease states, and various environmental factors [2, 3]. Notably, the deviation between predicted DNAm age and chronological age, known as epigenetic age acceleration, is associated with a wide-range of health and disease outcomes, including all-cause mortality [4], socioeconomic adversity [5] and smoking [6, 7], and metabolic outcomes, such as diabetes [8] and coronary artery disease (CAD) [9]. Consequently, DNA methylation-based clocks are posited as biomarkers for early disease risk and predictors of life expectancy and overall mortality. As epigenetic signatures can be modifiable [7], DNAm-based predictors hold great promise for clinical utilization. Nevertheless, the potential of DNAm-based predictors as a clinical tool remains untapped in the field of trauma, as the impact of physical trauma on biological aging, as measured by epigenetic drift, remains largely unexplored.

Burn injuries are among the most severe forms of trauma, affecting approximately nine million people worldwide each year, with over 110,000 deaths attributed to burn-related complications [10, 11]. Of greater concern is the escalating incidence of new burn cases and associated deaths in North America, owing to population growth and the rapidly aging population [12, 13]. While advances in acute clinical treatment have significantly improved survival rates and immediate outcomes for burn patients over the past few decades, emerging evidence reveals enduring health consequences persist for years after the initial injury. Indeed, several retrospective analyses of over 30,000 patients admitted with a burn injury between 1980-2012 revealed increased admissions for a broad range of age-related conditions, including cardiovascular disease [14], diabetes [15] and musculoskeletal conditions [16]. With that being said, it is not surprising that thermal injuries and various traumatic events are also well-established triggers of metabolic and inflammatory alterations that parallel those observed in aging and have been reported to drive and contribute to the aging process itself [17-19]. Indeed, burn injuries are known to provoke an intense and sustained hypermetabolic response, unmatched by other physiological stressors [20, 21]. Although a direct causal link between this response and epigenetic aging in burn patients has yet to be established, previous studies have associated hypermetabolic states and elevated energy expenditure with accelerated epigenetic aging, suggesting that such states may mirror the ‘metabolaging’ phenotype [22-25].

To further reinforce the link between aging and post-burn outcomes, severe burn injuries not only increase the risk of chronic age-related disease, but there is a growing body of literature supporting the notion that burn injuries may also decrease lifespan. A recent population-based cohort study demonstrated that burn survivors have a significantly higher rate of long-term mortality for at least 5-10 years after the injury compared to matched controls [26]. Additionally, the incidence and severity of long-term poor outcomes have been reported to increase with age. In fact, previous work has proved chronological age as an independent risk factor for death and poor long-term outcomes in patients with burn injuries, with older adult burn patients having a 3 to 5 fold increase in mortality compared to younger counterparts, even with similar burn size and the same incidence of inhalation injury [27-29]. Despite the urgent need to improve these long-term adverse outcomes, the precise mechanisms driving the enduring health consequences of burn injuries, including reduced lifespan and an elevated risk of early-onset age-related diseases, remain incompletely understood. These findings suggest that long-term mortality and medical morbidities may be clinical manifestations of an underlying accelerated cellular aging process potentiated by a burn injury. To date, however, little is known about the impact of burn injuries on epigenetic aging and whether this factor might help predict the risk of mortality and long-term poor outcomes.

Thus, in this study, we aimed to examine whether burn injuries are linked to accelerated cellular and biological aging, as indicated by epigenetic drift. Given recent evidence indicating that epigenetic age increases in response to life stressors and disease, we hypothesize that burn injuries similarly potentiate biological and cellular aging and that this accelerated epigenetic age is causally linked with poor long-term outcomes. To test this, we estimated the epigenetic age of whole blood in burn patients and healthy individuals using the previously established DNAm clocks (HorvathAge, GrimAge, PhenoAge, and DunedinPoAm). We defined epigenetic age acceleration for each case by comparing the individual’s epigenetic age with their chronological age to assess whether accelerated or dysfunctional epigenetic aging is associated with the onset and severity of the burn injury. We then measured how burn injuries affect the epigenetically inferred risk of mortality and frailty. Lastly, we corroborated our findings using a well-established murine model of thermal injury.

## MATERIALS AND METHODS

### Study Design, Participants, and Approval

This prospective cohort study enrolled burn patients admitted to two adult provincial burn centres in Ontario, Canada: The Ross Tilley Burn Centre at Sunnybrook Health Sciences Centre in Toronto, Ontario, between January 1, 2022, and July 31, 2022, and the Burn Unit at Hamilton Health Sciences, in Hamilton, Ontario, Canada, between January 1, 2023, and July 31, 2024. Non-burn healthy volunteers aged 18 years or older with no significant health issues were recruited as controls from both sites through study-related advertisements posted at the hospitals. Approval for this study was obtained from the Research Ethics Board of Sunnybrook Health Sciences Centre (#194-2010) and by the Hamilton Integrated Research Ethics Board (#15833). Written informed consent for prospective blood collection was received from participants before study inclusion.

Burn patients were selected based on the following inclusion criteria: ≥18 years of age, ≥2% total body surface area (TBSA), and required at least one burn-related operative procedure to manage their injuries. Patients admitted for reconstruction and frostbite were excluded. All patients received standard-of-care treatment for their injuries according to the established clinical protocols, which included early excision and grafting, early nutritional intervention, adequate antibiotic coverage, and ventilation, as needed. Whole blood samples were collected from burn patients on admission and up to twice weekly during hospitalization alongside clinical blood collection. Healthy volunteers provided a one-time donation of whole blood.

### Clinical Outcomes

Patient demographics and clinical outcomes were prospectively recorded by the burn team, including the attending physician and critical care staff. These include demographic data, injury characteristics, burn size, inhalation injury, pre-admission comorbidities, in-hospital mortality, organ failure, pneumonia, and sepsis, as defined by the ABA Guidelines [30]. The Revised Baux Score was calculated for each patient, as defined by Osler et al. [31]. Length of stay (LOS) was determined as the days elapsed between hospital admission and discharge from the Burn Unit.

### Animals and Thermal Injury Model

Animal experiments were conducted in accordance with the McMaster University Animal Research Ethics Board (AREB) (Hamilton, Ontario, Canada; AUP#22-08-30). Male wild-type C57BL/6J mice were purchased from Jackson Laboratories (Bar Harbor, Maine), housed at ambient temperature and cared for in accordance with the Guide for the Care and Use of Laboratory Animals. The aged mice were 75 weeks old (n = 11 mice). The environmental temperature was 21°C with a 12-hour light/dark cycle and food and water were available *ad libitum*. Before the burn injury, whole blood was collected using EDTA-coated capillary tubes. Then, all mice were anesthetized with 2.5% isoflurane and shaved along the dorsal spine region. Ringer’s lactate was injected in the dorsal region subcutaneously (2 mL) in all mice to protect the spine and intraperitoneally (0.5 mL) for resuscitation. Buprenorphine (0.05-0.1 mg/kg body weight) was administered for pain management. A full-thickness, third-degree scald burn encompassing 20% of TBSA was achieved by immersing the dorsum of the mice in 98-degree Celsius water for 10 seconds. Burned mice were subsequently housed individually in sterile cages and food and water were given *ad libitum*. All injured rodents were health scored by laboratory staff daily to minimize animal pain and distress. Health scoring was based on a scale of 15, with up to 3 points given for eyes and nose, activity, food intake, grooming, and hydration. Seven days following the injury, the mice were euthanized, and whole blood was collected and stored at -80 degrees Celsius until further analysis (Fig. 5A).

### DNAm Profiling of Biological Age and Epigenetic Clocks for Burn Patients

DNA was extracted from whole blood using a Qiagen DNeasy blood kit according to the manufacturer's protocol. To efficiently detect epigenetic signatures of aging, DNA samples were tested on a custom targeted methylation array. By systematically querying epigenetic databases (EWAS catalogue & datahub) and the literature for large (N>1000) epigenome-wide association studies (EWAS), ~50,000 CpG sites with robust associations to age-related traits were built into a custom Illumina Infinium methylation array. Raw methylation data (idats) were transformed to beta-values using the OpenSesame pipeline from the Sesame package (openSesame: The openSesame pipeline in sesame: Tools For Analyzing Illumina Infinium DNA Methylation Arrays). This function carries out normal-exponential out-of-band (NOOB) correction, nonlinear dye bias correction, and *P*-value assessment with out-of-band array hybridization (pOOBAH), utilizing a *P*-value threshold of 0.05. To ensure probe and sample quality, stepwise filtering for missing data was performed, alternating between probe and sample, with progressively stricter thresholds of 20%, 10%, and 5%. Epigenetic sex was assigned based on the ratio of median methylation levels between X-chromosome and Y-chromosome probes. Samples with discrepancies between epigenetic sex and reported sex, or those with inconclusive epigenetic sex, were excluded from the analysis. Quality control metrics for bisulfite conversion, hybridization, extension, and dye specificity were calculated using Illumina's preset controls. Samples that failed two or more of these tests at the recommended thresholds were excluded from the analysis.

Multiple biological clocks were derived, including those trained on (i) chronological age (HorvathAge) [32], (ii) mortality (GrimAge; predicts time to death by measuring methylation proxies for protein levels associated with mortality) [33], (iii) functional capacity or “healthspan” (PhenoAge; based on clinical markers and comorbidities that estimate phenotypic age) [6], and (iv) capturing aging trajectory (as opposed to the prior “snapshots” of biological age) based on longitudinal change in age-related protein biomarkers (Dunedin (P)ace(o)f(A)ging(m)ethylation; predicts the rate of age-related changes in physical and cognitive traits over one year) [34]. Frailty methylation risk score (MRS) was derived based on a 20-CpG marker score from Li *et al.* (2022) [35]. Mortality MRS was derived based on a 177 CpG score from Huan *et al.* (2022) [36]. MRS were standardized to have a mean of 0 and standard deviation of 1.

### Association Testing Between Burn Phenotypes and DNA Methylation for Burn Patients

As several patients had blood collected and tested at multiple timepoints (0-4, 5-14, 15-27, and 28+ days post-burn), the mean values for epigenetically inferred traits were taken as the representative value for each participant. The association between burn phenotypes (status, severity) and epigenetically inferred traits (DNAmclocks, MRS) was evaluated using linear regression. The burn phenotype was considered the primary independent variable, and the epigenetically inferred trait was the dependent variable. All regression analyses adjusted for chronological age and sex as covariates, except for stratified analyses by gender or age strata. Association testing was performed in R version 4.3.2 (R Foundation for Statistical Computing, Vienna, Austria).

### DNAm Profiling of Biological Age and Epigenetic Clocks for Burn Mice

DNA was extracted from whole blood specimens collected from mice before and 7-days after burn injury. DNA was bisulfite-converted and >285,000 CpG methylation markers were profiled using the Illumina Infinium Mouse Methylation Beadchip. Mouse methylation data was pre-processed using the *Sesame* R package with parameters configured according to mouse-specific recommendations by Zhou *et al.*) [37]. Further quality control to remove both CpG probes and samples with low detectability was applied (<90% probe and sample call rates). From the mouse methylation array data, we derived multi-tissue (Thompson *et al.*, 2018) [38] and liver-specific (Wang *et al.*, 2017) [39] mouse clocks using ClockBase [40].

**Table 1:** Demographics for all burn patients and healthy controls.

	Burns	Controls	*P*
**No. of Patients**	**59**	**25**	
**Demographics**			
Age, years, mean (SD)	48.6 (16.76)	41.36 (19.11)	0.115
Sex, n (%)			0.004
Males	37 (63%)	7 (28%)	
Females	22 (37%)	18 (72%)	
Body Mass Index, median (IQR)	22.36 (19.89-26.12)	19.78 (18.44-22.30)	0.084
Weight (kg), median (IQR)	76.50 (66.18-92.36)	69.00 (60.00-79.00)	0.046
Height (m), mean (SD)	1.70 (0.092)	1.67 (0.083)	0.383
**Injury Characteristics**			
Burn Etiology, n (%)			
Flame	35 (59%)		
Scald	11 (19%)		
Other	13 (22%)		
TBSA, median (IQR)	9.00 (4.00-20.00)		
TBSA 3^rd^ degree, median (IQR)	5.00 (0.00-16.38)		
Inhalation Injury, n (%)	9 (16%)		
Revised Baux Score, median (IQR)	58.50 (48.75-89.75)		
**Outcomes**			
LOS, days, median (IQR)^#^	16.50 (10.00-30.00)		
	n = 46		
30-Day Mortality, n (%)	1 (2%)		
**Complications**	n = 46		
Sepsis, n (%)	5 (9%)		
Pneumonia, n (%)	7 (12%)		

LOS, Length of Stay; TBSA, Total Body Surface Area.

# Analysis restricted to patients alive until discharge. Numbers may not add to 100 due to rounding

### Statistical Analysis

Data are reported in accordance with the Strengthening the Reporting of Observational Studies in Epidemiology (STROBE) statement [41]. Demographic data were analyzed using the Student’s *t*-test, Mann-Whitney *U* test, and Fisher's exact test, as appropriate. Timepoint analyses were conducted using the Kruskal-Wallis test. Heterogeneity was tested using Cochran’s *Q*. In the patient tests, all tests were two-tailed, with *P* <0.05 considered statistically significant. Mouse results were analyzed using one-tailed student’s t-tests, with *P* <0.05 considered statistically significant. Analyses were performed using SPSS Statistics version 29.0 (IBM Corporation, Armonk, NY), GraphPad Prism 10.0.0 (GraphPad Software, Boston, MA), and R version 4.3.2. Figures were generated using GraphPad Prism version 10.0.0 and R version 4.3.2.

## RESULTS

### Study Population Characteristics and Outcomes

To study the effect of burn injury on epigenetic aging, we conducted a DNA methylation study on whole blood collected from burn patients and healthy individuals. During the study period, 59 burn patients and 25 healthy controls were confirmed eligible and included in the final analysis. Table 1 describes the baseline characteristics of the study population. The burn patients did not differ from the healthy individuals in terms of age, with a mean (SD) of 48.6 (16.76) and 41.36 (19.11) years, respectively (*P*=0.12). Body mass index (BMI) was also similar between the burn (22.4 (19.9-26.1)) and control (19.8 (18.4-22.3)) groups (*P*=0.08), despite increased admission weight (kg) in the burn group (76.5 (66.2-92.4) vs. 69.0 (60.0-79.0); *P*=0.046). A greater proportion of males were in the burn group (63% vs. 28%; *P*=0.004).

**Figure 1.**
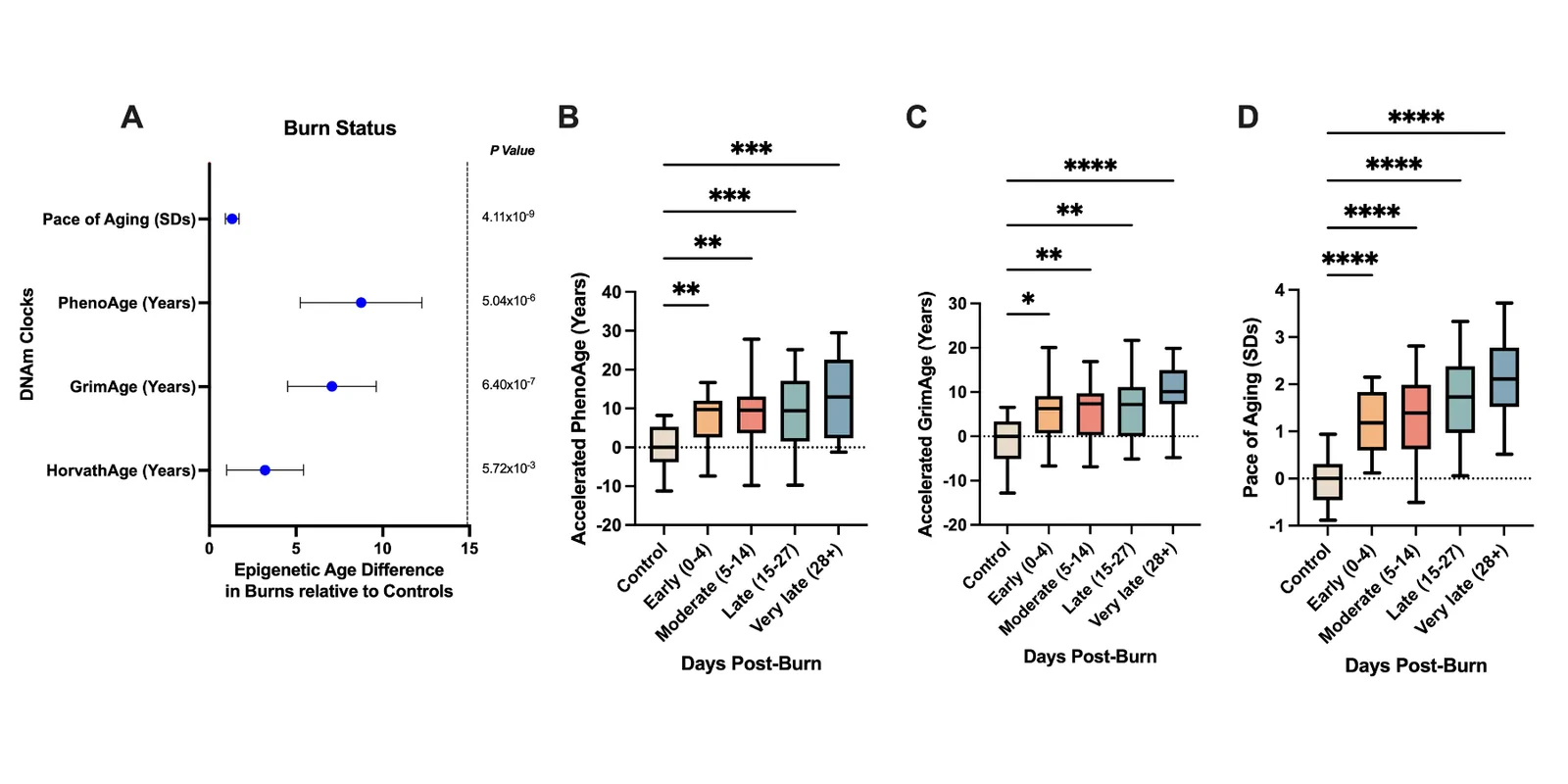
**Burn patients exhibit elevated epigenetic age acceleration compared to non-burn healthy controls**. (**A**) Forest plot demonstrating the epigenetic age difference between burn and non-burn patients calculated by various clocks (Pace of Aging (SDs), PhenoAge (years), GrimAge (years), and HorvathAge (years)), adjusted for chronological age and sex. Accelerated biological aging (Median (IQR)) of burn patients compared to controls at various timepoints following injury (0-4, 5-14, 15-27, and 28+ days post-burn) as calculated by (**B**) PhenoAge (years), (**C**) GrimAge (years), and (**D**) Pace of Aging (SDs). N_burn_ = 59; N_control_ = 25. *P<0.05, **P<0.01, ***P<0.001, ****P<0.0001. SDs - Standard deviations.

In terms of injury characteristics, the majority (59%) of burn patients experienced a flame-related injury, and 16% presented with an inhalation injury on admission. The median (IQR) burn size was 9.0% (4.0-20.0) TBSA, with a median (IQR) of 5.0% (0.0-16.4) full thickness. Regarding clinical outcome assessments, the median LOS was 16.5 (10.0-30.0) days, with a mortality of 2% at 30 days post-admission. The incidence of sepsis and pneumonia in-hospital were 9% and 12%, respectively.

### Burn Injuries Accelerate Epigenetic Aging, as Indicated by DNAm Clocks, and this Effect is Persistent in Patients

We initially assessed epigenetic biological aging in the whole blood of burn patients and healthy controls by calculating their DNAm age utilizing various second- and third-generation epigenetic clocks trained to measure healthspan and lifespan (GrimAge, PhenoAge, and DunedinPoAm), along with the first-generation Horvath clock trained to predict chronological age. All three clocks-HorvathAge (R^2^=0.90; *P*=1.49×10^-43^), GrimAge (R^2^=0.84; *P*=8.28×10^-35^), and PhenoAge (R^2^=0.82; *P*=2.24×10^-32^)-showed strong correlations with the chronological age of both burn patients and healthy controls, serving as a reliable positive control and confirmation that the methylation scores were appropriately derived (Supplementary Fig. 1).

Burn patients exhibited marked epigenetic age acceleration as compared to healthy controls, equivalent to 3.21 HorvathAge years (95% CI, 1.00-5.43; *P=* 5.72×10^-3^), 7.06 GrimAge years (95% CI, 4.51-9.63; *P=* 6.40×10^-7^), 8.75 PhenoAge years (95% CI, 5.25-12.25; *P=* 5.04×10^-6^), and a 1.32 standard deviation (SD) increase in the pace of aging as determined by DunedinPoAm (95% CI, 0.93-1.71; *P=* 4.11×10^-9^) (Fig. 1A). Notably, elevated epigenetic age acceleration persists from the early days post-burn (0-4 days; +6.29 GrimAge years; *P*_GrimAge_=0.02, +9.74 PhenoAge years; *P*_PhenoAge_=5.70×10^-3^, and +1.18 DunedinPoAm SDs; *P*_DunedinPoAm_ < 0.0001) through to the later time points (28+ days; +10.08 GrimAge years; *P*_GrimAge_ < 0.0001, +12.99 PhenoAge years; *P*_PhenoAge_= 2.0×10^-4^, and +2.11 DunedinPoAm SDs; *P*_DunedinPoAm_ < 0.0001), suggesting that the effect is also chronic (Fig. 1B-D). Together, these findings indicate that burn injuries accelerate epigenetic biological aging independently of age and sex, with the effect lasting for at least 28 days.

**Figure 2.**
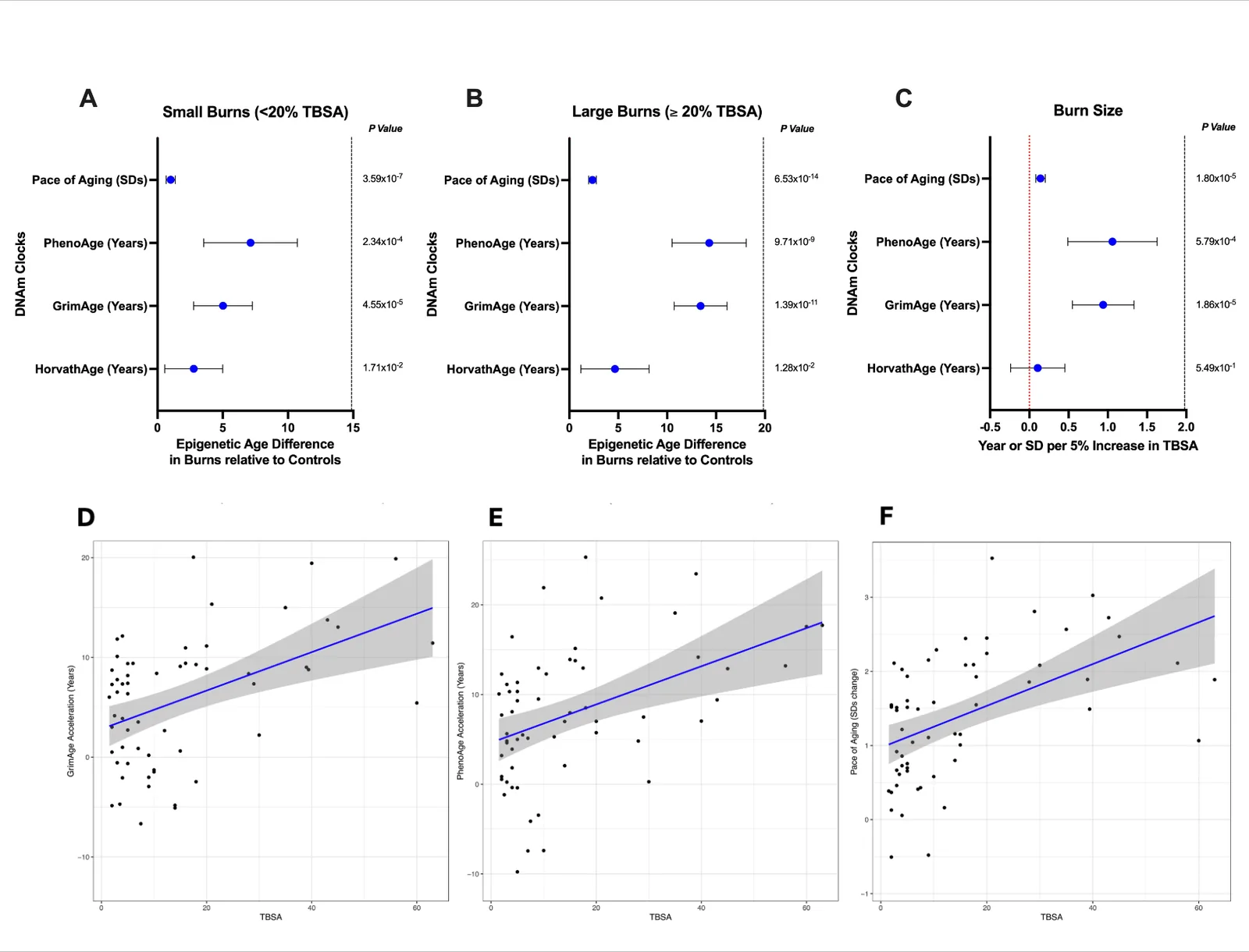
**Burn size correlates with the rate of the accelerated aging in burn patients**. Forest plot demonstrating the average epigenetic age difference between patients with (**A**) small burns (< 20% TBSA; N_burn_ = 44; N_control_ = 25) or (**B**) large burns (≥ 20% TBSA; N_burn_ = 15; N_control_ = 25) and non-burn patients calculated by various clocks (Pace of Aging (SDs), PhenoAge (years), GrimAge (years), and HorvathAge (years)). (**C**) The average year or SD increase per 5% increase in TBSA according to the respective DNAm clocks (N_burn_ = 59; N_control_ = 25). Linear regression of (**D**) GrimAge (years), (**E**) PhenoAge (years), (**F**) Pace of Aging (SDs change), against TBSA of all burn patients (N_burn_ = 59; N_control_ = 25). Adjusted for chronological age and sex. TBSA - total body surface area; SDs - Standard deviations.

### Accelerated Epigenetic Aging Observed After Burn Strongly Correlates with Burn Size in Patients

Next, we analyzed the association of burn injury severity with epigenetic aging. In general, burns affecting ≥20% of an adult's TBSA are classified as severe injuries and typically require hospitalization and extensive rehabilitation [17]. Studies have shown that, when compared to minor burns, patients with severe burns face considerably worse outcomes, including prolonged recovery times, greater susceptibility to infections, and an elevated risk of complications such as organ failure, mainly due to a profound and prolonged hypermetabolic response that persists for years after injury [17, 18, 20]. Therefore, we hypothesized that the impact of a burn injury on accelerated aging varies based on burn size. To test this, we stratified our patients into two groups: those with burns covering <20% TBSA (N_burn_=44; N_control_=25) and those with burns covering ≥20% TBSA (N_burn_=15; N_control_=25). The two subgroups showed no differences in age, sex, BMI, inhalation injury, 30-day mortality, or sepsis (Supplementary Table 1). However, the larger burn group had a higher Revised Baux Score (*P*<0.001) and increased incidence of pneumonia (*P=*0.012), which are expected outcomes for burns with greater TBSA (Supplementary Table 1).

**Figure 3.**
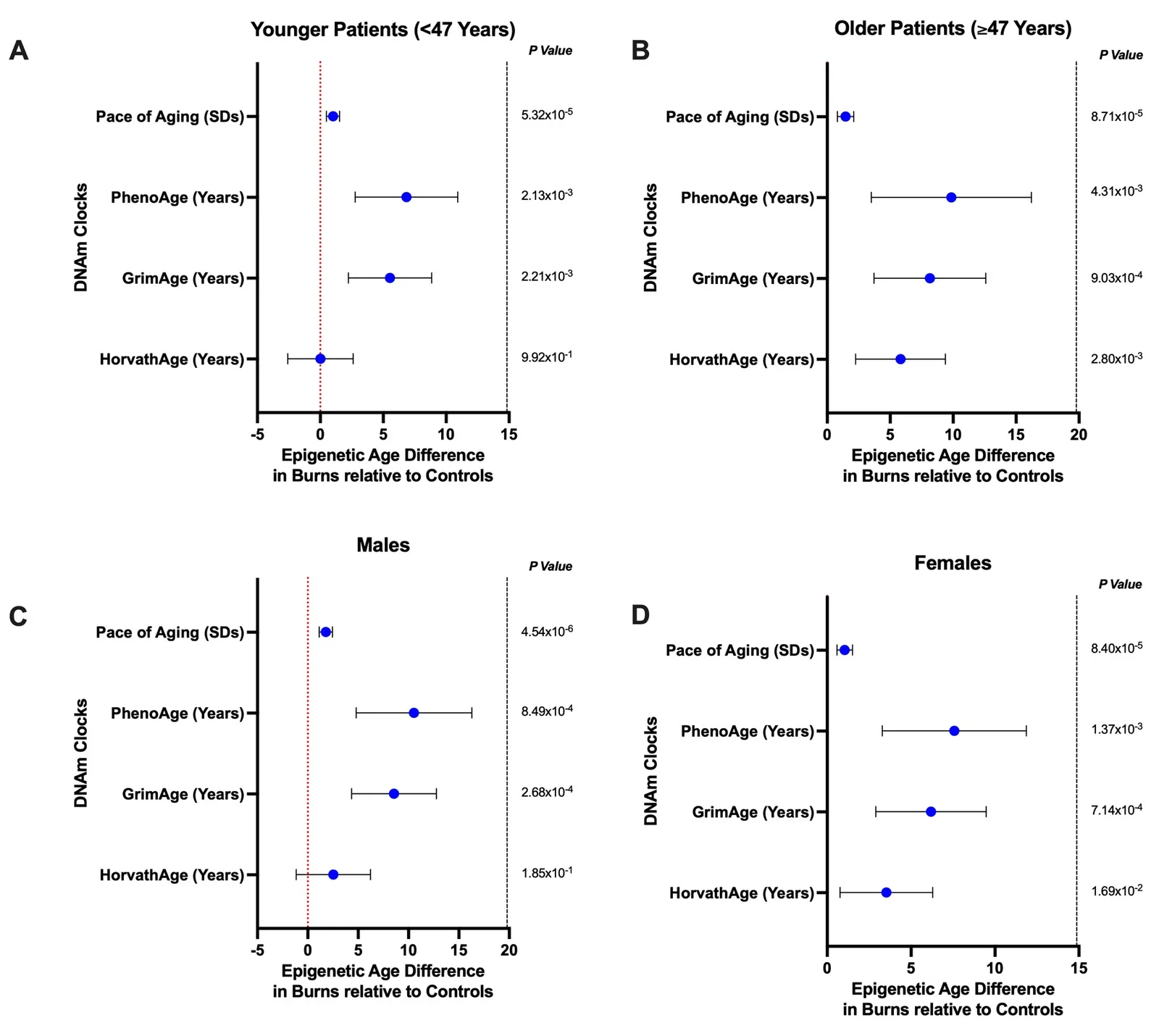
**Burn injuries accelerate biological aging across sexes and in both younger and older adult patients**. Forest plot demonstrating the average epigenetic age difference between (A) younger (< 47 years) (N_burn_ = 27; N_control_ = 15), (B) older (≥ 47 years) (N_burn_ = 32; N_control_ = 10) (C) male (N_burn_ = 30; N_control_ = 7), or (D) female (N_burn_ = 18; N_control_ = 18) burn patients and their respective non-burn patient controls calculated by various clocks (Pace of Aging (SDs), PhenoAge (years), GrimAge (years), and HorvathAge (years)). The age analyses are adjusted for sex, and the sex analyses are adjusted for chronological age. SDs - Standard deviations.

After adjusting for sex and age as covariates, patients with smaller burns exhibited an epigenetic age acceleration of 2.77 HorvathAge years (95% CI, 0.55-4.99; *P=* 1.71×10^-2^), 5.01 GrimAge years (95% CI, 2.77-7.27; *P=* 4.55×10^-5^), 7.13 PhenoAge years (95% CI, 3.54-10.72; *P=* 2.34×10^-4^), and 1.00 DunedinPoAm SDs (95% CI, 0.65-1.35; *P=* 3.59×10^-7^) (Fig. 2A). Patients with larger burns exhibited epigenetic age acceleration of 4.66 HorvathAge years (95% CI, 1.18-8.15; *P=* 1.28×10^-2^), 13.42 GrimAge years (95% CI, 10.71.0-16.13; *P=* 1.39×10^-11^), 14.29 PhenoAge years (95% CI, 10.51-18.08; *P=* 9.71×10^-9^), and 2.36 DunedinPoAm SDs (95% CI, 1.97-2.75; *P=* 6.53×10^-14^) (Fig. 2B). Consistent with our hypothesis, patients with larger burns exhibited a near 2 to 3-fold increase in epigenetic acceleration. In fact, there was significant heterogeneity between biological age differences among patients with minor burns and those with severe burns, with respect to GrimAge (*P*=2.93×10^-6^), PhenoAge (*P*=7.16×10^-3^), and DunedinPoAm (*P*=4.01×10^-7^), indicating the substantial variability in the results is attributable to differences in TBSA (Fig. 3A-B). HorvathAge (*P*=0.37) did not report heterogeneity.

Further supporting a direct role of the burn injury as an accelerant of aging, the severity of the burn was proportional to the magnitude of age acceleration (Fig. 2D-F). Indeed, we observed that for every 5% increase in TBSA, epigenetic age was accelerated 0.94 GrimAge years, or ~11 months (95% CI, 0.55-1.33; *P=* 1.86×10^-5^), 1.06 PhenoAge years, or ~13 months (95% CI, 0.49-1.63; *P=* 5.79×10^-4^), and 0.14 DunedinPoAm SDs (95% CI, 0.08-0.20; *P=* 1.80×10^-5^) (Fig. 2C). HorvathAge (+0.10 years; 95% CI, -0.24-0.45; *P*=0.55) showed no change per 5% TBSA increase. Overall, our data demonstrate a dose-dependent increase in epigenetic age acceleration that depends on the severity of the burn injury.

### Burns Accelerate Epigenetic Aging Across Biological Sexes and Age Groups in Patients

Having established a clear association between burn severity and epigenetic aging, we subsequently assessed the impact of age and sex, two well-established risk factors for death and disability due to injuries, on epigenetic aging acceleration following burn injury. To investigate this, we stratified both burn patients and healthy controls into two groups based on age: <47 years for the younger adults (N_burn_=27; N_control_=15) and ≥47 years for the older adults (N_burn_=32; N_control_=10) and adjusted for sex as a covariate. This age cutoff was chosen to ensure that both groups had adequate sample sizes to maintain sufficient statistical power. It was also based on a previous study showing that the post-burn stress response tends to diminish in individuals aged 50 and older, with this inability to mount an appropriate stress response being linked to increased mortality [42]. These two subgroups showed no significant differences in sex, BMI, TBSA, or inhalation injury on admission, and similar 30-day mortality, pneumonia, and sepsis (Supplementary Table 2). We then stratified our patients into two groups based on sex: males (N_burn_=30; N_control_=7) and females (N_burn_=22; N_control_=18) and adjusted for age as a covariate. There were no differences in relevant demographic information between males and females (Supplementary Table 3).

Older adult burn patients had an increase of 5.82 HorvathAge years (95% CI, 2.25-9.39; *P=* 2.80×10^-3^), 8.16 GrimAge years (95% CI, 3.72-12.59; *P=* 9.03×10^-4^), 9.86 PhenoAge years (95% CI, 3.49-16.22; *P=* 4.31×10^-3^), and 1.46 DunedinPoAm SDs (95% CI, 0.81-2.11; *P=* 8.71×10^-5^), compared to healthy older adult controls (Fig. 3B). In contrast, younger adult burn patients had an epigenetic age acceleration equivalent to 5.54 GrimAge years (95% CI, 2.23-8.85; *P=* 2.21×10^-3^), 6.85 PhenoAge years (95% CI, 2.78-10.92; *P=* 2.13×10^-3^), and 1.01 DunedinPoAm SDs (95% CI, 0.49-1.53; *P=* 5.32×10^-4^), compared to healthy adult controls (Fig. 3A). In younger burn patients, HorvathAge (+0.01 years; 95% CI, -2.58-2.61; *P=* 9.92×10^-1^) did not demonstrate an acceleration. There was significant heterogeneity between biological age differences among adult and older adult burn patients, with respect to Horvath’s clock (*P*=0.01) and GrimAge (*P*=0.04) indicating that the substantial variability in the results is attributable to inter-age differences (Fig. 3A-B). PhenoAge (*P*=0.43) and DunedinPoAm (*P*=0.29) reported no significant heterogeneity.

Moreover, although HorvathAge (+2.54 years; 95% CI, -1.15-6.23; *P=* 0.19) showed no significant increase, burn injuries accelerated the epigenetic age of males by 8.56 GrimAge years (95% CI, 4.35-12.77; *P=* 2.68×10^-4^), 10.54 PhenoAge years (95% CI, 4.80-16.28; *P=* 8.49×10^-4^), and 1.79 DunedinPoAm SDs (95% CI, 1.12-2.45; *P=* 4.54×10^-6^) (Fig. 3C). Conversely, female burn patients experienced accelerated epigenetic aging by 3.52 HorvathAge years (95% CI, 0.76-6.29; *P=* 1.69×10^-2^), 6.19 GrimAge years (95% CI, 2.90-9.47; *P=* 7.14×10^-4^), 7.58 PhenoAge years (95% CI, 3.29-11.87; *P=* 1.37×10^-3^), and 1.04 DunedinPoAm SDs (95% CI, 0.58-1.50; *P=* 8.40×10^-5^) (Fig. 3D). None of the clocks exhibited significant heterogeneity between males and females (*P*>0.05 for all). Together, we showed that burns accelerate aging across different age groups and sexes demonstrating that this effect is consistent among all burn patients. Moreover, there are no differences in post-burn acceleration between males and females; however, older adults exhibit significantly greater age acceleration following a burn injury compared to younger burn patients.

### Burns Increase Epigenetically Predicted Risks of Mortality and Frailty in Patients

**Figure 4.**
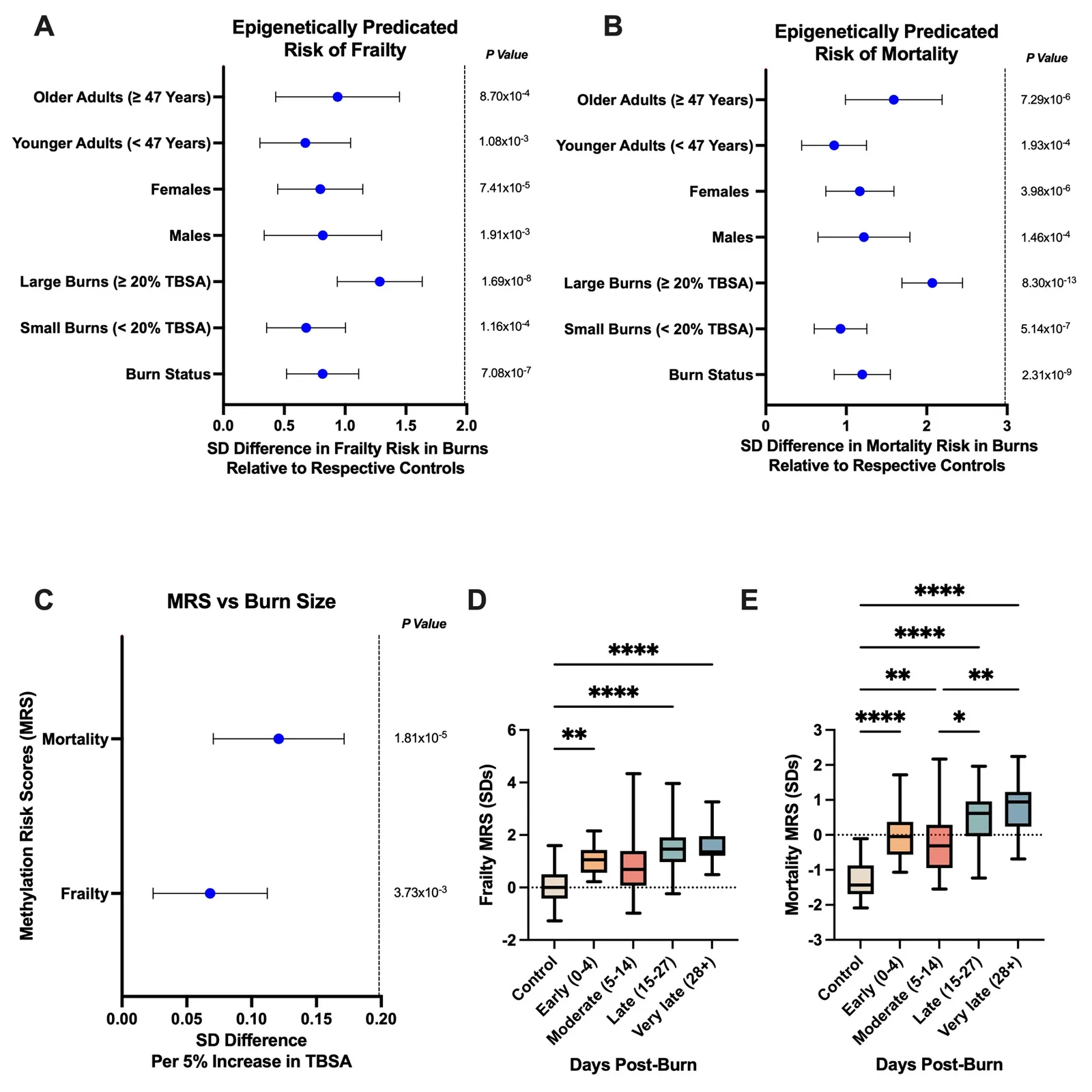
**Burn patients have increased epigenetically inferred risks of mortality and frailty**. Forest plots demonstrating the difference in SDs of methylation risk scores of **(A)** frailty and **(B)** mortality in all subgroups of burn patients (older adults, younger adults, females, males, larger burn, smaller burns, and overall burn status), compared with their respective healthy controls. (**C**) The average increase in methylation risk scores (frailty and mortality) per 5% increase in TBSA of all burn patients. (**D**) frailty and (**E**) mortality methylation risk scores in controls and burn patients over time (0-4, 5-14, 15-27, and 28+ days post-burn). Sample size: N_burn_ = 59; N_control_ = 25; older adults (N_burn_ = 27; N_control_ = 15); younger adults (N_burn_ = 27; N_control_ = 15); females (N_burn_ = 18; N_control_ = 18); males (N_burn_ = 30; N_control_ = 7); large burns (N_burn_ = 15; N_control_ = 25); small burns (N_burn_ = 44; N_control_ = 25). TBSA - total body surface area; SDs - Standard deviations; MRS - Methylation risk scores.

Beyond biological aging, DNAm scores can also capture risk for comorbidities and disease outcomes, such as frailty and mortality, providing a glimpse into future clinical risk. Interestingly, burn patients’ epigenetically determined risks of frailty (+0.81 SDs; 95% CI, 0.52-1.11; *P*=7.08×10^-7^) and mortality (+1.20 SDs; 95% CI, 0.85-1.55; *P*=2.31×10^-9^) were significantly higher than controls, suggesting substantially greater future risk of disability and death (Fig. 4A-B). More intriguingly, both small (Frailty MRS: +0.68 SDs; 95% CI, 0.35-1.00; *P*=1.16×10^-4^, Mortality MRS: +0.93 SDs; 95% CI, 0.60-1.26; *P*=5.14×10^-7^) and large burns (Frailty MRS: +1.29 SDs; 95% CI, 0.94-1.63; *P*=1.69×10^-8^, Mortality MRS: +2.07 SDs; 95% CI, 1.69-2.45; *P*=8.30×10^-13^) raise the risks of mortality and frailty (Fig. 4A-B). Moreover, our findings indicate that for every 5% increase in TBSA affected, the epigenetically inferred risk of mortality rises by 0.12 SDs (95% CI, 0.07-0.17; *P*= 1.81×10^−5^), while the risk of frailty increases by 0.07 SDs (95% CI, 0.02-0.11; *P*= 3.73×10^−3^) (Fig. 4C). This aligns with the literature indicating that TBSA is a significant prognostic indicator of disability and outcomes in burn patients [17, 43], as well as with our findings showing a strong correlation between TBSA and epigenetic age acceleration.

**Figure 5.**
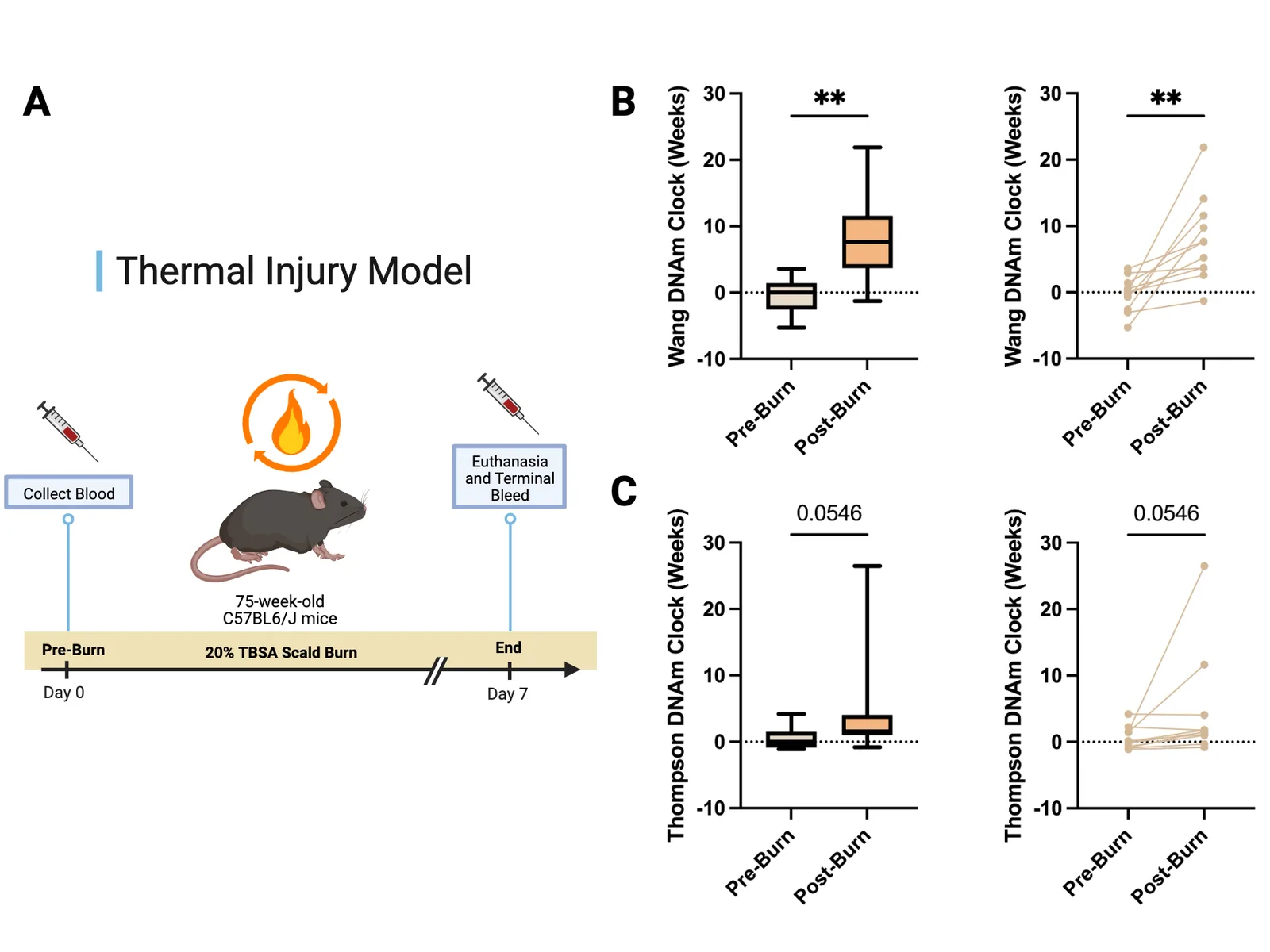
**Aged burn mice exhibit an elevated epigenetic age 7-days post-burn compared to before the burn injury**. (**A**) Schematic demonstrating the animal study timeline. The average age (weeks) acceleration between the post-burn and pre-burn time points according to the (**B**) Wang DNA methylation clock and the (**C**) Thompson DNA methylation clock. Time points were compared using one-tailed t-test. N = 11 mice, **P<0.01.

Additionally, after adjusting for chronological age as a covariate, both males (Frailty MRS: +0.82; 95% CI, 0.33-1.30; *P*=1.91×10^-3^, Mortality MRS: +1.22; 95% CI, 0.65-1.79; *P*=1.46×10^-4^) and females (Frailty MRS: +0.80; 95% CI, 0.45-1.15; *P*=7.41×10^-5^, Mortality MRS: +1.17; 95% CI, 0.75-1.59; *P*=3.98×10^-6^) exhibited a similar increase in epigenetically determined risks of mortality and frailty (Fig. 4A-B). Similarly, both older adults (Frailty MRS: +0.94; 95% CI, 0.43-1.45; *P*=8.70×10^-4^, Mortality MRS: +1.59; 95% CI, 0.99-2.19; *P*=7.29×10^-6^) and younger adults (Frailty MRS: +0.67; 95% CI, 0.30-1.05; *P*=1.08×10^-3^, Mortality MRS: +0.85; 95% CI, 0.45-1.25; *P*=1.93×10^-4^) exhibited significant increases in the predicted risks of frailty and mortality following a burn injury (Fig. 4A-B).

Even more intriguingly, the increases in epigenetically predicted risks of mortality and frailty seem to persist over time, much like the sustained rise in epigenetic age acceleration that we observed. Indeed, the predicted risk of mortality and frailty are elevated from the early days post-burn (0-4 days; +1.06 frailty MRS SDs; *P*_Frailty_ < 0.01, -0.05 mortality MRS SDs; *P*_Mortality_ < 0.0001) through to the later time points (28+ days; +1.36 frailty MRS SDs; *P*_Frailty_ < 0.0001, +0.94 mortality MRS SDs; *P*_Mortality_ < 0.0001) (Fig. 4D-E). Collectively, our results show that burn injuries elevate the epigenetically predicted risk of mortality and frailty in a dose-dependent manner, regardless of age and sex, with this increase lasting for up to 28 days after the injury.

### Burn Injuries Accelerate Epigenetic Aging in Mice

Lastly, we utilized our well-established murine model of thermal injury to investigate the causal relationship between burn injuries and biological aging. Given that older adult burn patients are the most susceptible to the aging effects of burn injuries, we selected aged mice for our methylation study. By comparing matched blood samples collected before the burn injury and seven days post-injury (equivalent to ~280 human days), we observed that burn injuries accelerated aging by 3.99 mouse weeks according to the Thompson Clock (*P* = 0.054) and 8.17 weeks according to the Wang Clock (*P* = 1.7 × 10^-3^) (Fig. 5B-C). After adjusting for the seven days that elapsed between blood draws, burn injuries accelerated aging by 3.22 mouse weeks based on the Thompson Clock (*P* = 0.094) and 7.40 weeks based on the Wang Clock (*P* = 3.0 × 10^-3^). This corresponds to 2.5 and 5.8 human years of aging, respectively, aligning with the accelerated aging observed in our patient population.

## DISCUSSION

Aging is a dynamic and complex biological process influenced by various factors, including genetics, lifestyle, environmental exposures, and pathological conditions, all of which can accelerate the progression of cellular and systemic decline [44]. However, the impact of burn injuries on the biological aging process remains underexplored. It has been well-established that burn injuries trigger a cascade of metabolic alterations, impacting systemic inflammatory, endocrine, autonomic, and immune pathways and resulting in immense physiological derangement [17]. While initially ubiquitous and essential for maintaining organ function and whole-body homeostasis under demanding conditions, the hypermetabolic stress response to burn injury is unrivalled in terms of its magnitude and duration, frequently persisting for up to 3 years post-injury to the detriment of patients [20]. Evidently, this prolonged post-burn hypermetabolism includes changes that not only mirror those observed in aging but have also been reported to drive and contribute to the aging process itself [18, 19]. These changes include chronic systemic inflammation, compromised mitochondrial function, heightened oxidative stress, multi-organ dysfunction, alterations in body composition and fat distribution, and increased frailty [17, 45, 46]. Given that both aging and burn-induced hypermetabolism share multiple pathogenic mechanisms, it is plausible that systemic, molecular, and cellular changes resulting from burn injuries drive the premature onset of aging phenotypes throughout survivorship, ultimately leading to increased long-term morbidity and mortality in burn patients due to accelerated biological aging. Nevertheless, while existing literature unequivocally establishes the interconnection of aging, disease, and biological stress, the impact of burn injuries on biological aging represents a substantial knowledge gap.

Here, we addressed this critical gap by testing the hypothesis that a burn injury contributes to long-term poor health outcomes by accelerating biological aging. Indeed, all well-established methylation clocks indicated a strong correlation with an increasing acceleration of epigenetic aging in the blood samples of burn patients compared to healthy controls, even after adjusting for age and sex. In fact, severe burns (≥20% TBSA) accelerated aging by 13.42 years according to GrimAge and 14.29 years according to PhenoAge, making severe burn injuries the current most significantly known accelerant of epigenetic aging. This effect is nearly double that of chronic long-term smoking, which accelerates aging by approximately 7.91 years [33, 47]. Most notably, unlike other external stressors such as pregnancy, severe COVID-19 infection, and emergency surgery, which have been shown to induce a transient increase in epigenetic age that normalizes within days or weeks, our long-term epigenetic profiling further revealed that the observed effects persist for up to 28 days post-injury, also underscoring burn injuries as one of the most chronic accelerants of biological aging identified [2]. This was further supported by our murine model of thermal injury, which demonstrated that burn injuries accelerated epigenetic aging by an estimated equivalent of three to six human years. We speculate that this chronic effect could be associated with the persistence of the post-burn hypermetabolic response, suggesting that this accelerated aging could potentially last for years after the injury. However, additional longitudinal studies are needed to evaluate this. Together, our findings position burn injuries as the most significant and chronic accelerant of biological aging observed to date.

However, given that social and economic factors like low income, non-white ethnicity, and unemployment increase burn injury risk, along with the high prevalence of mental disorders such as alcohol use disorders in burn patients, it raises the question of whether burn injuries themselves or these factors—also recognized contributors to epigenetic aging—are the true culprits behind the accelerated aging [48, 49]. To strengthen the argument for burns as a direct accelerator of aging, we show that the size of the burn correlates with the degree of biological age acceleration. In fact, our findings indicate that for every 5% increase in TBSA burned, burn patients not only experience approximately one year of biological aging but also experience an increase in the pace at which they are aging. Interestingly, higher TBSA burns have also been shown to directly correlate with the severity of the hypermetabolic response to burn trauma, burn-related complications, comorbidities, and overall risk of mortality, mirroring the pattern observed in epigenetic age acceleration [17, 50, 51]. Given that diseases characterized by accelerated epigenetic aging often manifest an aging phenotype, as observed in Down's syndrome [52], obesity [53], and HIV infection [54], this data suggests that long-term mortality and medical morbidities associated with profound and prolonged burn-induced hypermetabolism may be clinical manifestations of an underlying accelerated cellular aging process potentiated by a severe burn injury.

Consequently, the accelerated epigenetic aging observed in burn patients may explain why they experience higher rates of chronic disease, reduced lifespan, and increased mortality within years following the initial injury [14, 15, 26]. However, it is important to note that not all survivors of burn injuries develop these complications, and it is desirable to detect patients who are at high risk for long-term adverse outcomes early to enable personalized treatment and prevention strategies. For instance, older adult burn patients have significantly worse outcomes compared to their younger counterparts, experiencing nearly a fourfold increase in mortality compared to younger adults, despite having similar burn sizes and inhalation injuries [27]. The underlying reasons for these poor outcomes in older patients remain unknown. However, we show that older adults exhibit approximately an 8 to 10-year acceleration in biological age, nearly 1.5-folds higher than younger adults, along with an accelerated pace of aging, which we speculate could be the tipping point, given that their bodies are already physiologically compromised and have a limited capacity to respond to the trauma [27, 29, 55, 56]. Interestingly, a recent study by Yamashita et al. found that basal methylation levels are crucial for age-related hypermethylation, suggesting that older adults may already be predisposed to the biological aging phenotype, which could amplify the age-accelerating effects of burn injuries [57]. Thus, accelerating the preexisting hallmark dysfunction that is inherent to aging by nearly a decade may overwhelm their capacity to cope. Of note, both males and females experienced similar burn-induced accelerated biological aging. However, the impact of sex differences on post-burn outcomes remains unclear, highlighting the need for further studies to explore sex-specific consequences of burn-induced aging.

Furthermore, in line with accelerated aging, our findings indicate that burn injuries also elevate the epigenetically predicted risk of mortality and frailty across all subgroups. Notably, these risks increased proportionally with the TBSA affected by the burn. This aligns with previous studies showing that burn survivors often face issues such as fatigue, reduced resistance, impaired ambulation, and a higher incidence of frailty and mortality after discharge [26, 46]. Notably, the MRS for both mortality and frailty remain elevated for up to 28 days following the burn injury, highlighting the chronic nature of this increased risk. These findings further underscore the long-term post-burn sequelae that burn survivors experience after discharge and the chronic effects of burn injury. Thus, by assigning an accurate risk score and identifying patients at the greatest risk for premature death and secondary medical morbidities early, we would enable more personalized treatment plans and implement targeted prevention strategies. In this regard, DNAm-based predictors may hold great promise for clinical utilization since epigenetic signatures can be modifiable [7].

Although the precise mechanistic pathways linking burn-induced pathologies to epigenetic aging, and vice versa, remain unclear, recent research has begun to shed light on these complex interactions. A recent study by Kabacik et al. investigating the hallmarks of aging and their role in the epigenetic aging profile identified impaired nutrient sensing, mitochondrial dysfunction, and stem cell exhaustion as the primary contributors [58]. Notably, both dysregulated nutrient sensing and mitochondrial dysfunction are hallmark features of the post-burn hypermetabolic response, suggesting that the hypermetabolic response may exacerbate epigenetic aging through these processes [17, 24, 59, 60]. Additionally, as noted earlier, hypermetabolic states and elevated energy expenditure have been linked to the acceleration of epigenetic aging, offering further evidence that they may play a critical role in contributing to this process [22, 23]. Conversely, this may not be a one-way relationship. Differential DNA methylation patterns have been shown to regulate gene expression, with increased methylation in the promoter region generally leading to gene silencing, while methylation within the gene body can enhance gene expression [61, 62]. This gene-level regulation may play a role in modulating and prolonging the hypermetabolic response and thus contributing to the onset of post-burn pathology, particularly if epigenetic changes are disrupting key pathways involved in inflammation, nutrient sensing, and other critical processes. Indeed, aberrant DNA methylation has been previously linked to the induction of hyperinflammation in cancer, which similar to burn injuries, manifests a hypermetabolic phenotype [63]. However, further research is needed to elucidate the mechanistic relationship between these epigenetic modifications and burn-induced pathology, specifically whether the post-burn hypermetabolic state drives epigenetic changes or if epigenetic alterations themselves are responsible for the burn-induced pathology.

Nevertheless, despite the significant insights provided by our study, several limitations need to be addressed in future research. Our study had a small overall sample size, with even fewer participants in stratified analyses by age and sex, particularly within the control group, which limits statistical power and warrants caution in interpreting subgroup findings. Additionally, while we acknowledge the potential impact of socioeconomic and psychosocial factors on DNA methylation patterns, we were unable to quantitatively assess or adjust for these variables due to limitations in available data. The absence of this information may confound the observed associations with epigenetic aging, potentially leading to discrepancies between our findings and the true underlying biological processes. Moreover, our study lacked access to medical records for the control group, preventing us from accounting for potential confounding factors such as prior surgeries, comorbidities, or other medical events that could influence their epigenetic aging.

To establish the full scientific validity of these findings, a larger and more controlled trial with a greater sample size is essential, including socioeconomic status-, age-, ethnicity-, and sex-matched controls to ensure that confounding factors are minimized, and that behavioral and psychosocial influences can be more rigorously accounted for. These larger and well-characterized studies should also prioritize investigating the associations between epigenetic aging and clinical complications and outcomes such as infections, delirium, mobility scores, sarcopenia indices, and disease severity scores (e.g., SOFA), particularly given the limitations in clinical data available in our study. Future work should also consider comparisons with other types of traumatic injuries, elective and emergent surgeries, and incorporate frailty scores, which may serve as important modifiers of epigenetic aging trajectories—either contributing to accelerated aging or reflecting a ceiling effect in already frail individuals. Altogether, these steps will be essential for understanding the clinical relevance of epigenetic biomarkers and their potential role in risk stratification and personalized care.

Moreover, our study only examined the acute effects of burn injuries on biological aging up to 28 days post-injury, leaving a gap in understanding how long these effects persist. Longitudinal studies, with extended follow-up periods, are required to determine the duration and potential long-term consequences of accelerated aging after burn trauma. Specifically, future studies should include three-year follow-ups, as the hypermetabolic response has been shown to persist for this duration, to determine whether accelerated aging follows a similar trajectory [20]. Additionally, future research should explore the biological pathways involved and seek to correlate these findings with actual clinical markers of frailty and mortality, rather than relying solely on epigenetic scores. The use of other biomarker analyses, including proteomics clocks, telomere length assessments, and additional robust biological markers, could help elucidate how these epigenetic alterations impact the genome and proteome. Understanding how these changes manifest at different molecular levels will provide a more comprehensive view of the biological aging process in burn survivors and its potential role in their long-term health outcomes. These directions will be critical to fully understanding the implications of burn-induced accelerated aging.

In summary, our study positions burn injuries as the most prominent and long-lasting accelerators of biological aging identified thus far. Understanding whether aging is accelerated post-burn not only opens new possibilities for improving long-term outcomes in burn patients but also provides insights for managing and preventing complications that affect the quality of life for all burn survivors. This study lays the foundation for uncovering the mechanisms linking burn injuries to premature aging, with the potential to revolutionize long-term care and outcomes for burn survivors, ultimately helping to reduce the future healthcare burden. While our results are based on burn injuries, the observed patterns of epigenetic aging may generalize to other forms of trauma due to shared features of systemic inflammation, physiological stress, and immune dysregulation. This underscores the profound implications for predicting recovery trajectories, guiding personalized interventions, and improving long-term outcomes across diverse critically injured populations.

## Supplementary Materials

The Supplementary data can be found online at: www.aginganddisease.org/EN/10.14336/AD.2025.0407.


