# Supplementary material for: Burn Injuries Accelerate Biological Aging and Increase the Epigenetically Inferred Risk of Mortality and Frailty
Source: Aging Dis. 2025 May 11;17(4):2198–212. doi: 10.14336/AD.2025.0407 (PMC13256645; doi:10.14336/AD.2025.0407)
Supplement: Supplementary file 1 [file AD-17-4-2198-s.pdf]

SUPPLEMENTARY DATA

Burn Injuries Accelerate Biological Aging and Increase the Epigenetically Inferred Risk of Mortality and Frailty

Fadi Khalaf, Serena Yang, Dalia Barayan, Diana Julia Tedesco, Michael Chong, Guillaume Paré, Marc G. Jeschke

© 2025. Khalaf F et al. Published online at http://www.aginganddisease.org/EN/10.14336/AD.2025.0407

SUPPLEMENTARY DATA

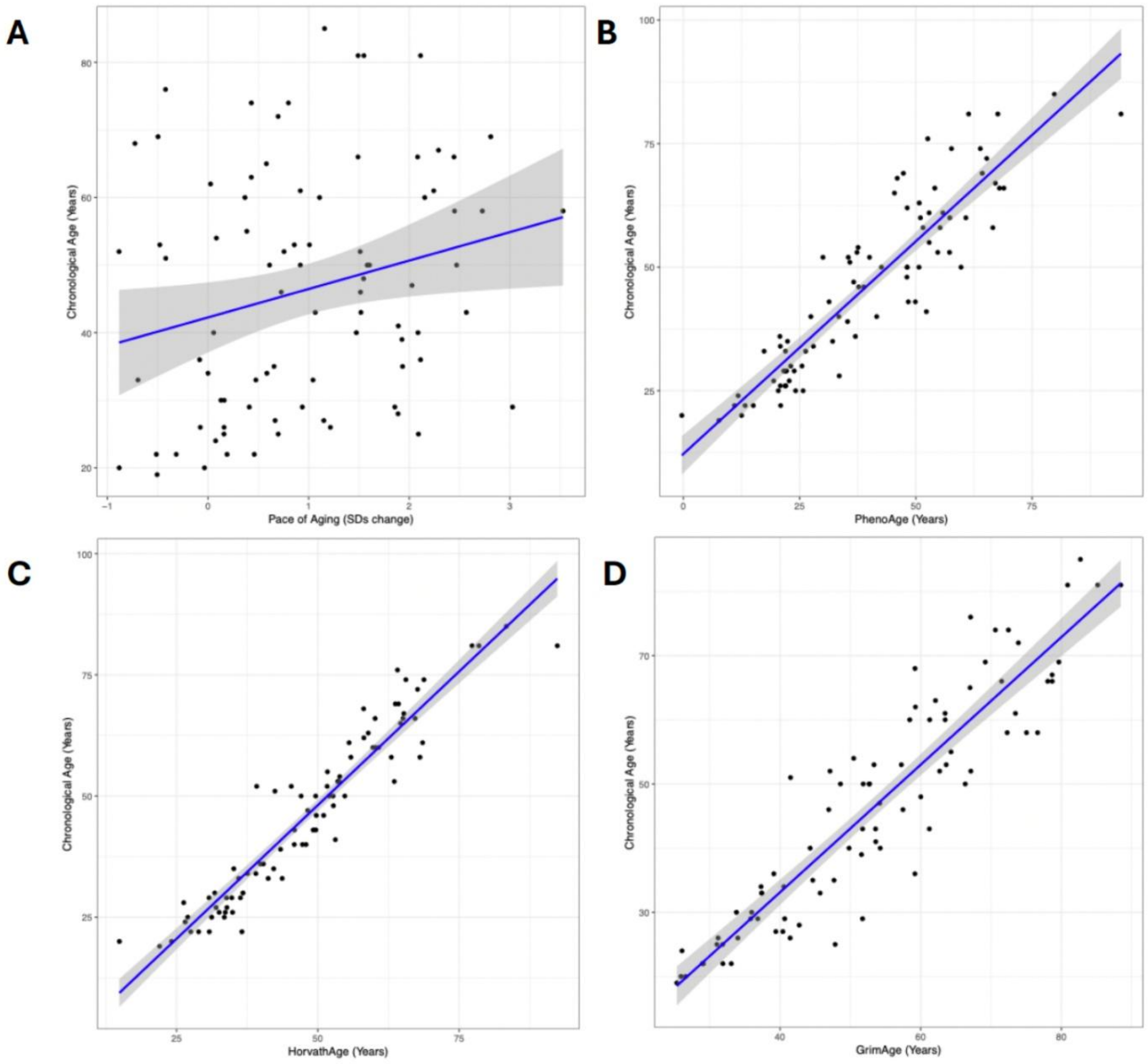

Supplementary Figure 1. DNA methylation age significantly correlated with the chronological age of all patients in the whole blood. Linear regression of (A) pace of Aging (SDs) calculated via DunedinPoAm, (B) PhenoAge (years), (C) HorvathAge (years), and GrimAge (years) against chronological age (years) of healthy controls and burn patients. N_{burn} = 59; $N_{control}$ = 25. SDs – Standard deviations.

© 2025. Khalaf F et al. Published online at http://www.aginganddisease.org/EN/10.14336/AD.2025.0407

SUPPLEMENTARY DATA

Supplementary Table 1. Demographics for all burn patients included in the TBSA sub-group analysis.

	TBSA <20%	TBSA≥20%	*P*
**No. of Patients**	**44**	**15**	
**Demographics**			
Age, years, mean (SD)	48.81 (17.47)	47.50 (15.17)	0.779
Sex, *n (%)*			0.558
Males	28 (65%)	9 (56%)	
Females	15 (35%)	7 (44%)	
Body Mass Index, mean (SD)	23.80 (6.61)	22.09 (3.19)	0.295
Weight (kg), median (IQR)	77.10 (63.88-94.06)	73.80 (67.93-81.60)	0.463
Height (m), mean (SD)	1.70 (0.09)	1.69 (0.09)	0.796
**Injury Characteristics**			
Burn Etiology, *n (%)*			
Flame	22 (51%)	13 (81%)	0.043
Scald	8 (19%)	3 (19%)	1.000
Other	13 (30%)	0 (0%)	0.012
TBSA, median (IQR) (SD)	5.00 (3.00-10.50)	39.00 (28.00-45.00)	<0.001
TBSA 3rd degree, median (IQR)	2.00 (0.00-6.00)	24.50 (19.50-43.00)	<0.001
Inhalation Injury, *n (%)*	4 (9%)	5 (31%)	0.052
Revised Baux Score, median (IQR)	55.00 (42.50-69.00)	96.00 (80.00-105.40)	<0.001
Outcomes			
LOS, days, median (IQR)[#]	20.63 (19.11)	45.00 (21.25)	0.036
	N = 38	*N = 8*	
30-Day Mortality, *n (%)*	0 (0%)	1 (13%)	0.174
**Complications**	*N = 38*	*N = 8*	
Sepsis, *n (%)*	3 (8%)	2 (25%)	0.203
Pneumonia, *n (%)*	3 (8%)	4 (50%)	0.012

LOS, Length of Stay; TBSA, Total Body Surface Area.
[#]Analysis restricted to patients alive until discharge.
Numbers may not add to 100 due to rounding

© 2025. Khalaf F et al. Published online at http://www.aginganddisease.org/EN/10.14336/AD.2025.0407

SUPPLEMENTARY DATA

Supplementary Table 2. Demographics for all burn patients included in the age sub-group analysis.

	Younger Adults (<47 years)	Older Adults (≥47 years)	P
**No. of Patients**	**27**	**32**	
**Demographics**			
Age, years, mean (SD)	33.56 (7.72)	61.03 (10.92)	<0.001
Sex, n (%)			0.788
Males	16 (59%)	21 (66%)	
Females	11 (41%)	11 (34%)	
Body Mass Index, mean (SD)	23.30 (7.44)	23.65 (4.59)	0.856
Weight (kg), median (IQR)	75.00 (59.35-88.45)	77.20 (68.24-92.72)	0.622
Height (m), mean (SD)	1.70 (0.10)	1.69 (0.09)	0.576
**Injury Characteristics**			
Burn Etiology, n (%)			
Flame	16 (59%)	19 (59%)	1.000
Scald	6 (22%)	5 (16%)	0.739
Other	5 (19%)	8 (25%)	0.754
TBSA, median (IQR)	6.50 (3.75-29.75)	13.50 (4.00-19.50)	0.975
TBSA 3rd degree, median (IQR)	5.00 (1.00-27.00)	2.50 (0.00-14.00)	0.201
Inhalation Injury, n (%)	5 (19%)	4 (13%)	0.719
Revised Baux Score, median (IQR)	44.50 (38.25-60.00)	76.65 (4.00-19.50)	<0.001
**Outcomes**			
LOS, days, median (IQR)[#]	17.50 (10.00-21.25)	16.50 (9.00-31.25)	0.724
	N = 22	N = 24	
30-Day Mortality, n (%)	0 (0%)	1 (4%)	1.000
**Complications**	N = 22	N = 24	
Sepsis, n (%)	2 (9%)	3 (13%)	1.000
Pneumonia, n (%)	4 (18%)	3 (13%)	0.694

LOS, Length of Stay; TBSA, Total Body Surface Area.
[#]Analysis restricted to patients alive until discharge.
Numbers may not add to 100 due to rounding

© 2025. Khalaf F et al. Published online at http://www.aginganddisease.org/EN/10.14336/AD.2025.0407

SUPPLEMENTARY DATA

Supplementary Table 3. Demographics for all burn patients included in the sex sub-group analysis.

	**Males**	**Females**	**P**
**No. of Patients**	**37**	**22**	
**Demographics**			
Age, years, mean (SD)	49.54 (17.87)	46.64 (14.94)	0.506
Body Mass Index, mean (SD)	23.46 (6.53)	23.50 (5.62)	0.983
Weight (kg), median (IQR)	75.80 (68.45-96.62)	80.30 (56.70-87.30)	0.904
Height (m), mean (SD)	1.73 (0.09)	1.64 (0.06)	<0.001
**Injury Characteristics**			
Burn Etiology, n (%)			
Flame	23 (62%)	12 (55%)	0.594
Scald	4 (11%)	7 (32%)	0.081
Other	10 (27%)	3 (14%)	0.334
TBSA, median (IQR)	10.00 (3.50-19.00)	7.00 (4.00-25.00)	0.821
TBSA 3rd degree, median (IQR)	5.00 (0.00-20.00)	4.00 (0.00-11.00)	0.299
Inhalation Injury, n (%)	7 (19%)	2 (9%)	0.461
Revised Baux Score, median (IQR)	62.00 (44.00-25.00)	57.00 (50.50-84.75)	0.758
**Outcomes**			
LOS, days, median (IQR)[#]	16.00 (10.25-31.50)	20.00 (9.25-28.50)	0.912
	N = 29	N = 17	
30-Day Mortality, n (%)	0 (0%)	1 (6%)	0.370
**Complications**	N = 29	N = 17	
Sepsis, n (%)	2 (7%)	3 (18%)	0.343
Pneumonia, n (%)	4 (14%)	3 (18%)	1.000

LOS, Length of Stay; TBSA, Total Body Surface Area.
[#]Analysis restricted to patients alive until discharge.
Numbers may not add to 100 due to rounding

© 2025. Khalaf F et al. Published online at http://www.aginganddisease.org/EN/10.14336/AD.2025.0407
